# Association of the longitudinal trajectory of urinary albumin/creatinine ratio in diabetic patients with adverse cardiac event risk: a retrospective cohort study

**DOI:** 10.3389/fendo.2024.1355149

**Published:** 2024-04-29

**Authors:** Hui Li, Yajuan Ren, Yongguang Duan, Peng Li, Yunfei Bian

**Affiliations:** ^1^ Department of Cardiology, First Hospital of Shanxi Medical University, Taiyuan, China; ^2^ Department of Cardiology, Second Hospital of Shanxi Medical University, Taiyuan, China; ^3^ Beijing Hospital, National Center of Gerontology, Institute of Geriatric Medicine, Chinese Academy of Medical Sciences, Beijing, China

**Keywords:** urinary albumin/creatinine ratio, trajectory, type 2 diabetes mellitus, major adverse cardiorenovascular events, association

## Abstract

**Objective:**

The baseline urinary albumin/creatinine ratio (uACR) has been proven to be significantly associated with the risk of major adverse cardiac events (MACE). However, data on the association between the longitudinal trajectory patterns of uACR, changes in glycated hemoglobin A1c (HbA1c), and the subsequent risk of MACE in patients with diabetes are sparse.

**Methods:**

This is a retrospective cohort study including 601 patients with type 2 diabetes mellitus (T2DM; uACR < 300 mg/g) admitted to The First Hospital of Shanxi Medical University and The Second Hospital of Shanxi Medical University from January 2015 to December 2018. The uACR index was calculated as urinary albumin (in milligrams)/creatinine (in grams), and latent mixed modeling was used to identify the longitudinal trajectory of uACR during the exposure period (2016–2020). The deadline for follow-up was December 31, 2021. The primary outcome was the MACE [a composite outcome of cardiogenic death, hospitalization related to heart failure (HHF), non-fatal acute myocardial infarction, non-fatal stroke, and acute renal injury/dialysis indications]. The Kaplan–Meier survival analysis curve was used to compare the risk of MACE among four groups, while univariate and multivariate Cox proportional hazards models were employed to calculate the hazard ratio (HR) and 95% confidence interval (CI) for MACE risk among different uACR or HbA1c trajectory groups. The predictive performance of the model, both before and after the inclusion of changes in the uACR and HbA1c, was evaluated using the area under the receiver operating characteristic (ROC) curve (AUC).

**Results:**

Four distinct uACR trajectories were identified, namely, the low-stable group (uACR = 5.2–38.3 mg/g, *n* = 112), the moderate-stable group (uACR = 40.4–78.6 mg/g, *n* = 229), the high-stable group (uACR = 86.1–153.7 mg/g, *n* = 178), and the elevated-increasing group (uACR = 54.8–289.4 mg/g, *n* = 82). In addition, five distinct HbA1c trajectories were also identified: the low-stable group (HbA1c = 5.5%–6.8%, *n* = 113), the moderate-stable group (HbA1c = 6.0%–7.9%, *n* = 169), the moderate-decreasing group (HbA1c = 7.4%–6.1%, *n* = 67), the high-stable group (HbA1c = 7.7%–8.9%, *n* = 158), and the elevated-increasing group (HbA1c = 8.4%–10.3%, *n* = 94). Compared with the low-stable uACR group, patients in the high-stable and elevated-increasing uACR groups were more likely to be older, current smokers, and have a longer DM course, higher levels of 2-h plasma glucose (PG), HbA1c, N-terminal pro-B-type natriuretic peptide (NT-proBNP), uACR, and left ventricular mass index (LVMI), while featuring a higher prevalence of hypertension and a lower proportion of β-receptor blocker treatment (*p* < 0.05). During a median follow-up of 45 months (range, 24–57 months), 118 cases (19.6%) of MACE were identified, including 10 cases (1.7%) of cardiogenic death, 31 cases (5.2%) of HHF, 35 cases (5.8%) of non-fatal acute myocardial infarction (AMI), 18 cases (3.0%) of non-fatal stroke, and 24 cases (4.0%) of acute renal failure/dialysis. The Kaplan–Meier survival curve showed that, compared with that in the low-stable uACR group, the incidence of MACE in the high-stable (HR = 1.337, 95% CI = 1.083–1.652, *p* = 0.007) and elevated-increasing (HR = 1.648, 95% CI = 1.139–2.387, *p* = 0.009) uACR groups significantly increased. Similar results were observed for HHF, non-fatal AMI, and acute renal injury/dialysis indications (*p* < 0.05). The multivariate Cox proportional hazards models indicated that, after adjusting for potential confounders, the HRs for the risk of MACE were 1.145 (*p* = 0.132), 1.337 (*p* = 0.007), and 1.648 (*p* = 0.009) in the moderate-stable, high-stable, and elevated-increasing uACR groups, respectively. In addition, the HRs for the risk of MACE were 1.203 (*p* = 0.028), 0.872 (*p* = 0.024), 1.562 (*p* = 0.033), and 2.218 (*p* = 0.002) in the moderate-stable, moderate-decreasing, high-stable, and elevated-increasing groups, respectively. The ROC curve showed that, after adding uACR, HbA1c, or both, the AUCs were 0.773, 0.792, and 0.826, which all signified statistically significant improvements (*p* = 0.021, 0.035, and 0.019, respectively).

**Conclusion:**

A long-term elevated uACR is associated with a significantly increased risk of MACE in patients with diabetes. This study implies that regular monitoring of uACR could be helpful in identifying diabetic patients with a higher risk of MACE.

## 1 Introduction

Type 2 diabetes mellitus (T2DM) increases the risk of adverse cardiac events (1). Many studies have demonstrated that the level of proteinuria measured by the urinary albumin/creatinine ratio (uACR) is an important prognostic indicator for major adverse cardiorenovascular events (MACE) and death in patients with T2DM (2). Studies have demonstrated that a uACR reduction of 30% per year is associated with a hazard ratio (HR) of −0.7 for the clinical outcome of chronic kidney disease (CKD) progression (3), and an increased level of the baseline uACR has been proven to be significantly associated with a higher risk of MACE (4). On the other hand, changes in albuminuria are individually used as surrogate endpoints in clinical trials of CKD progression and are strongly associated with treatment effects on clinical endpoints (5, 6). Although the relationship between a change in uACR and the progression of kidney disease is strong and consistent, data on the impact of the longitudinal patterns of uACR on the MACE risk in patients with diabetes are sparse (7). Therefore, we aimed to explore the association between longitudinal uACR trajectories, changes in glycated hemoglobin A1c (HbA1c), and the MACE risk in patients with T2DM.

## 2 Participants

### 2.1 Study design

This is a retrospective cohort study including 601 patients with T2DM (uACR < 300 mg/g) admitted to The First Hospital of Shanxi Medical University and The Second Hospital of Shanxi Medical University from January 2015 to December 2018. The uACR index was calculated as the urinary albumin (in milligrams)/creatinine (in grams), and latent mixed modeling was used to identify the trajectory of uACR during the exposure period (2016–2020). This study meets the ethical requirements of medical research and has been approved of The Medical Ethics Committee of First Hospital of Shanxi Medical University and Second Hospital of Shanxi Medical University. As this study is retrospective, informed consent from patients is exempt.

### 2.2 Inclusion and exclusion criteria

The inclusion criteria were 1) aged 18–85 years, with no restriction on gender; 2) T2DM was diagnosed based on the Chinese Diabetes Diagnosis and Treatment Guidelines (8), including typical symptoms of diabetes (i.e., polydipsia, polyuria, polydipsia, and unexplained weight loss), as well as random blood glucose ≥11.1 mmol/L, fasting blood glucose (FBG) ≥7.0 mmol/L, or 2-h oral glucose tolerance test plasma glucose (PG) ≥11.1 mmol/L, or HbA1c ≥6.5%; 3) with baseline uACR <300 mg/g; 4) with complete uACR data examined during the exposure period (2016–2020); and 5) important baseline clinical characteristics and follow-up data were not missing.

The exclusion criteria were as follows: 1) complicated with acute heart failure at 6 months before admission; 2) complicated with acute myocardial infarction, severe heart failure [New York Heart Association (NYHA) class IV], or stroke at 6 months before admission; 3) complicated with valvular heart disease or congenital heart disease; and 4) complicated with tumors, with the expected survival period being less than 1 year.

### 2.3 Methods

By searching the electronic case database of our hospital, we recorded the demographic data (age and sex); risk factors and/or comorbidities (current smoker, diabetes history, hypertension, and hyperlipidemia); laboratory test results [FBG, 2-h postprandial PG, HbA1c (examined in 2016–2020), N-terminal pro-B-type natriuretic peptide (NT-proBNP), and low-density lipoprotein cholesterol (LDL-C)]; uACR levels (examined in 2016–2020), cardiac ultrasound results [left ventricular ejection fraction (LVEF), left ventricular end-diastolic and end-systolic diameters, and left ventricular mass index (LVMI)]; hypoglycemic drugs [insulin, metformin, and sodium–glucose co-transporter 2 inhibitor (SGLT-2i)]; glucagon-like peptide-1 receptor agonist (GLP-1 RA); and cardiovascular drugs [β-receptor blockers, angiotensin-converting enzyme inhibitors (ACEIs)/angiotensin II receptor blockers (ARBs), spironolactone, statins, anti-platelets, and anticoagulants].

### 2.4 Follow-up and end point events

Based on the patients’ follow-up data, the deadline for follow-up was set for December 31, 2021. The primary outcome was MACE [a composite outcome of cardiogenic death, hospitalization related to heart failure (HHF), non-fatal acute myocardial infarction, non-fatal stroke, and acute renal injury/dialysis indication].

The definition of heart failure was based on the following: 1) symptoms or signs of heart failure (e.g., dyspnea, palpitations, fatigue, and edema, among others); 2) LVEF < 50%; and 3) NT-proBNP > 450 ng/L (<50 years), >900 ng/L (50–75 years), and >1,800 ng/L (>75 years) (9).

The definition of acute myocardial infarction was based on the patients’ clinical symptoms, including chest pain, changes in the electrocardiogram (ECG), and elevated myocardial injury markers (e.g., cardiac troponin I, cTNI) (10).

Stroke includes both hemorrhagic and ischemic strokes, most of which are ischemic. The diagnostic criteria were 1) acute onset; 2) focal neurological deficits manifested as weakness, numbness, or language impairment on one side of the face or limbs, with a few presenting as comprehensive neurological deficits; 3) responsible lesions detected by MRI; and 4) excluding non-vascular causes (11).

The diagnosis of acute kidney injury was based on the 2021 Global Guidelines for Improving the Prognosis of Kidney Disease, and met one of the following three criteria (12): 1) an increase in serum creatinine >26.5 μmol/L (0.3 mg/dL) within 48 h; 2) an increase in serum creatinine to more than 1.5 times the upper limit of the reference range (men, 53–106 μmol/L; women, 44–97 μmol/L), and is known or suspected to occur within 7 days; and 3) urine volume <0.5 mL kg^−1^ h^−1^, course >6 hours. Indications for dialysis included 1) hyperkalemia beyond drug control (blood potassium >6.5 mmol/L); 2) water sodium retention, oliguria, anuria, and high edema complicated by heart failure, pulmonary edema, or hypertension; 3) severe metabolic acidosis (pH < 7.2); and 4) complicated by uremic pericarditis, pleurisy, central nervous system symptoms such as trance, drowsiness, coma, and convulsions, and psychiatric symptoms (13).

### 2.5 Statistical methods

STATA 12.0 was used for data analysis. The mean ± standard deviation was used to represent quantitative data with normal distribution, and one-way ANOVA was applied for inter-group comparisons. The median (Q1–Q3) was used to describe the quantitative data with a non-normal distribution, and Wilcoxon’s rank-sum test was applied for inter-group comparison. Case numbers and percentages represented qualitative data, and the chi-square test was applied for inter-group comparisons. In addition, the Kaplan–Meier survival curve was applied to compare the long-term follow-up MACE risk among the four uACR trajectory groups. The Cox regression model was used to evaluate the association between the uACR trajectory groups and MACE risk and to calculate the HR and 95% CI. The predictive performance of the model, both before and after the inclusion of changes in the uACR and the HbA1c trajectory groups, was evaluated using the area under the receiver operating characteristic (ROC) curve (AUC). A bilateral test was performed, with *p* < 0.05 indicating a statistically significant difference.

## 3 Results

### 3.1 uACR and HbA1c trajectories

**Figure 1:**
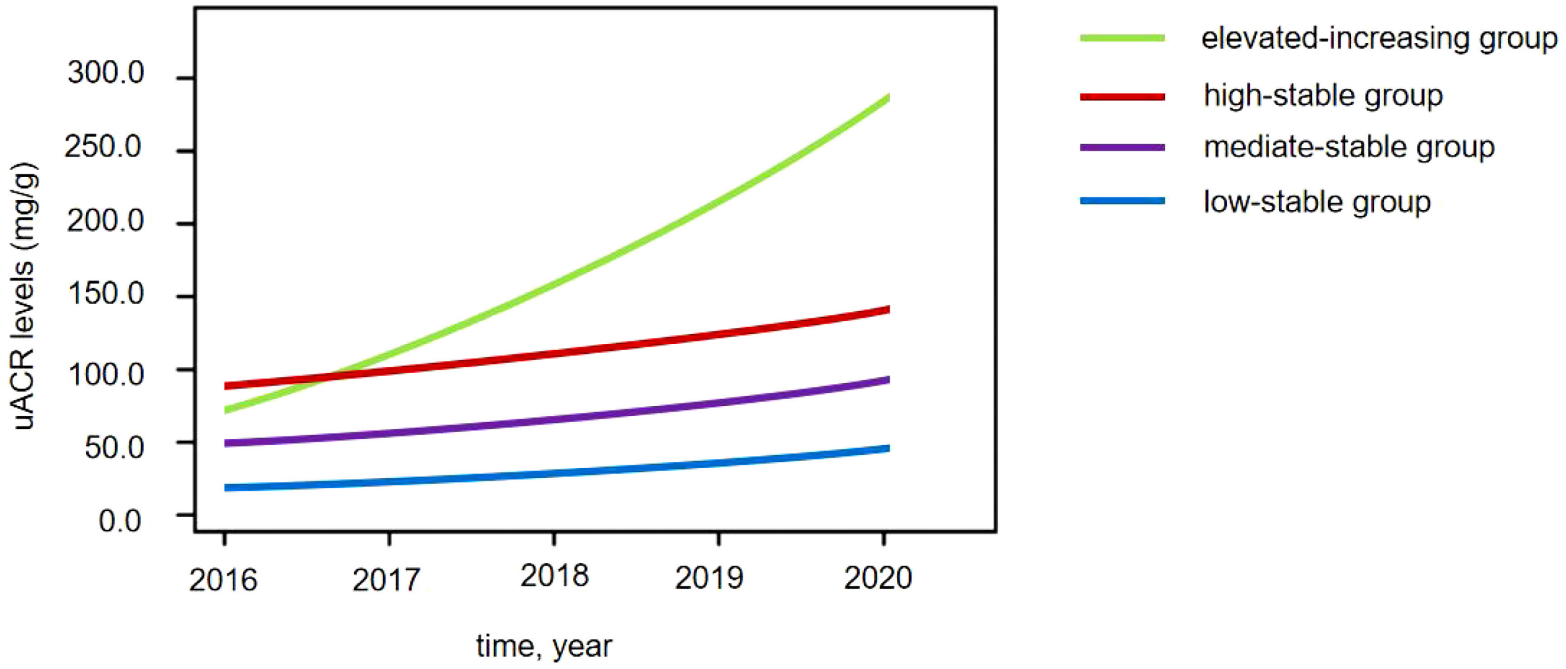
Four distinct urinary albumin/creatinine ratio (uACR) trajectories in patients with diabetes.

Four distinct uACR trajectories were identified during 2016–2020: the low-stable group (uACR = 5.2–38.3 mg/g, *n* = 112), the moderate-stable group (uACR = 40.4–78.6 mg/g, *n* = 229), the high-stable group (uACR = 86.1–153.7 mg/g, *n* = 178), and the elevated-increasing group (uACR = 74.8–289.4 mg/g, *n* = 82) (**Figure 1**).

In addition, five distinct HbA1c trajectories were also identified: the low-stable group (HbA1c = 5.5%–6.8%, *n* = 113), the moderate-stable group (HbA1c = 6.0%–7.9%, *n* = 169), the moderate-decreasing group (HbA1c = 7.4%–6.1%, *n* = 67), the high-stable group (HbA1c = 7.7%–8.9%, *n* = 158), and the elevated-increasing group (HbA1c = 8.4%–10.3%, *n* = 94).

### 3.2 Baseline characteristics of the four uACR trajectories

Compared with to the low-stable group, patients in the high-stable and elevated-increasing groups were more likely to be older, current smokers, and have had a longer DM course and have higher levels of 2-h PG, HbA1c, NT-proBNP, uACR, and LVMI, while featuring a higher prevalence of hypertension and a lower proportion of β-receptor blocker treatment (*p* < 0.05) (**Table 1**).

**Table 1:** Baseline characteristics of the four urinary albumin/creatinine ratio (uACR) trajectories.

Variable	Total (*n* = 601)	Low-stable group (*n* = 112)	Moderate-stable group (*n* = 229)	High-stable group (*n* = 178)	Elevated-increasing group (*n* = 82)	*p*-value
Age (years)	56.2 ± 18.5	53.9 ± 17.4	54.6 ± 14.2	57.1 ± 14.3	58.3 ± 16.5	0.084
Men, *n* (%)	358 (59.6)	64 (57.1)	138 (60.3)	103 (57.9)	53 (64.6)	0.704
Current smoker, *n* (%)	219 (36.4)	37 (33.0)	91 (39.7)	57 (32.0)	34 (41.5)	0.258
DM course (years)	8.32 ± 2.54	7.41 ± 1.33	8.25 ± 1.78	9.42 ± 2.20	10.51 ± 2.83	<0.001
Hypertension, *n* (%)	246 (40.9)	42 (37.5)	81 (35.4)	80 (44.9)	43 (52.4)	0.027
Hyperlipidemia, *n* (%)	270 (44.9)	46 (41.1)	100 (43.7)	83 (46.6)	41 (50.0)	0.597
FBG (mmol/L)	7.84 ± 2.05	7.54 ± 2.02	7.61 ± 1.71	7.65 ± 2.13	7.73 ± 2.46	0.927
2-h PG (mmol/L)	12.7 ± 2.8	11.9 ± 1.7	12.1 ± 2.1	12.6 ± 2.6	13.4 ± 3.3	<0.001
HbA1c 2016 (%)	7.72 ± 1.63	7.54 ± 1.09	7.68 ± 0.82	7.85 ± 1.12	8.05 ± 1.31	0.003
HbA1c 2017 (%)	7.81 ± 2.07	7.62 ± 1.13	7.77 ± 0.69	7.93 ± 0.91	8.24 ± 1.05	0.025
HbA1c 2018 (%)	7.89 ± 1.83	7.63 ± 1.02	7.83 ± 0.80	7.98 ± 1.06	8.32 ± 1.40	0.016
HbA1c 2019 (%)	7.94 ± 2.14	7.70 ± 1.23	7.91 ± 0.87	8.13 ± 1.27	8.38 ± 1.04	0.007
HbA1c 2020 (%)	8.12 ± 2.43	7.75 ± 1.18	7.98 ± 0.74	8.22 ± 1.22	8.45 ± 1.21	0.011
NT-proBNP (ng/L)	275.3 ± 65.2	262.1 ± 51.6	271.6 ± 40.4	283.0 ± 70.7	292.4 ± 76.5	0.001
LDL-C (mmol/L)	3.34 ± 0.56	3.25 ± 0.49	3.31 ± 0.34	3.40 ± 0.57	3.33 ± 0.62	0.071
uACR in 2016 (mg/g)	53.7 ± 21.6	20.0 ± 5.3	51.9 ± 13.2	97.8 ± 20.3	74.1 ± 11.7	<0.001
uACR in 2017 (mg/g)	63.1 (38.2–102.7)	20.5 ± 5.8	54.2 ± 14.6	107.2 ± 15.2	103.5 ± 18.4	<0.001
uACR in 2018 (mg/g)	76.3 (46.8–126.3)	23.8 ± 5.4	63.5 ± 14.1	112.1 ± 18.6	154.2 ± 29.5	<0.001
uACR in 2019 (mg/g)	138.2 (67.4–169.5)	29.0 ± 7.6	73.8 ± 16.7	120.5 ± 29.2	225.6 ± 43.7	<0.001
uACR in 2020 (mg/g)	144.1 (60.2–184.0)	28.4 ± 8.1	70.6 ± 15.2	123.7 ± 23.5	238.3 ± 50.1	<0.001
LVEF (%)	54.7 ± 6.3	55.3 ± 5.4	54.8 ± 4.8	54.2 ± 6.5	53.6 ± 7.7	0.175
LVESD (mm)	36.2 ± 4.6	35.7 ± 4.1	36.0 ± 3.7	36.4 ± 5.3	37.0 ± 6.2	0.221
LVEDD (mm)	47.1 ± 6.8	46.6 ± 5.3	46.9 ± 5.0	47.4 ± 7.5	47.9 ± 8.3	0.462
LVMI (g/m^2^)	138.4 ± 17.5	134.2 ± 13.4	136.1 ± 10.2	138.7 ± 17.3	138.4 ± 22.1	0.057
Insulin, *n* (%)	219 (36.4)	43 (38.4)	76 (33.2)	67 (37.6)	33 (40.2)	0.603
Metformin, *n* (%)	163 (27.1)	25 (22.3)	63 (27.5)	52 (29.2)	23 (28.0)	0.625
SGLT-2i, *n* (%)	136 (22.6)	31 (27.7)	52 (22.7)	38 (21.3)	15 (18.3)	0.372
GLP-1 RA, *n* (%)	98 (16.3)	22 (19.6)	43 (18.8)	32 (18.0)	12 (14.6)	0.822
β-receptor blocker, *n* (%)	103 (17.1)	25 (22.3)	40 (17.5)	30 (16.9)	8 (9.8)	0.152
ACEI/ARB, *n* (%)	134 (22.3)	23 (20.5)	50 (21.8)	40 (22.5)	21 (25.6)	0.861
Spironolactone, *n* (%)	31 (5.2)	5 (4.5)	11 (4.8)	10 (5.6)	5 (6.1)	0.941
Statins, *n* (%)	207 (34.4)	39 (34.8)	75 (32.8)	63 (35.4)	30 (36.6)	0.911
Anti-platelets, *n* (%)	155 (25.8)	25 (22.3)	58 (25.3)	50 (28.1)	22 (26.8)	0.737
Anticoagulants, *n* (%)	83 (13.8)	13 (11.6)	31 (13.5)	28 (15.7)	11 (13.4)	0.793

*DM*, diabetes mellitus; *FBG*, fasting blood glucose; *PG*, plasma glucose; *HbA1c*, hemoglobin A1c; *NT-proBNP*, N-terminal pro-B-type natriuretic peptide; *LDL-C*, low-density lipoprotein cholesterol; *LVEF*, left ventricular ejection fraction; *LVESD*, left ventricular end-systolic diameter; *LVEDD*, left ventricular end-diastolic diameter; *LVMI*, left ventricular mass index; *SGLT-2i*, sodium–glucose co-transporter 2 inhibitor; *GLP-1 RA*, glucagon-like peptide-1 receptor agonist; *ACEI*, angiotensin-converting enzyme inhibitors; *ARB*, angiotensin II receptor blockers.

### 3.3 MACE risk in the four uACR trajectories

During a median follow-up of 45 months (range, 24–57 months), 118 cases (19.6%) of MACE were identified, including 10 cases (1.7%) of cardiogenic death, 31 cases (5.2%) of HHF, 35 cases (5.8%) of non-fatal AMI, 18 cases (3.0%) of non-fatal stroke, and 24 cases (4.0%) of acute renal failure/dialysis. The Kaplan–Meier survival curve showed that, compared with that in the low-stable group, the incidence of MACE in the high-stable group (HR = 1.337, 95% CI = 1.083–1.652, *p* = 0.007) and in the elevated-increasing group (HR = 1.648, 95% CI = 1.139–2.387, *p* = 0.009) significantly increased. Similar results were observed for HHF, non-fatal AMI, and acute renal injury/dialysis indications (*p* < 0.05), but significant associations were not found between uACR trajectory and risk of cardiogenic death or non-fatal stroke (*p* > 0.05) (**Table 2**).

**Table 2:** Risk of major adverse cardiac events (MACE) in the four urinary albumin/creatinine ratio (uACR) trajectories.

MACE	Total (*n* = 601)	Low-stable group (*n* = 112)	Moderate-stable group (*n* = 229)	High-stable group (*n* = 178)	Elevated-increasing group (*n* = 82)	*p*-value
Cardiogenic death, *n* (%)	10 (1.7)	2 (1.8)	3 (1.3)	3 (1.7)	2 (2.4)	0.157
HHF, *n* (%)	31 (5.2)	4 (3.6)	10 (4.4)	11 (6.2)	6 (7.3)	0.026
Non-fatal AMI, *n* (%)	35 (5.8)	5 (4.5)	12 (5.2)	11 (6.2)	7 (8.5)	0.038
Non-fatal stroke, *n* (%)	18 (3.0)	3 (2.7)	7 (3.1)	5 (2.8)	3 (3.7)	0.195
Acute renal injury/dialysis indications, *n* (%)	24 (4.0)	3 (2.7)	7 (3.1)	9 (5.1)	5 (6.1)	0.021
All MACE, *n* (%)	118 (19.6)	17 (15.2)	39 (17.0)	39 (21.9)	23 (28.0)	0.024

HHF, hospitalization for heart failure; AMI, acute myocardial infarction.

### 3.4 Cox proportional hazards models

The multivariate Cox proportional hazards models indicated that age (HR = 1.545, *p* = 0.019), course of diabetes (HR = 1.436, *p* = 0.003), HbA1c (HR = 1.548, *p* = 0.008), NT-proBNP (HR = 1.764, *p* = 0.006), LVMI (HR = 1.461, *p* = 0.005), SGLT-2i (HR = 0.832, *p* = 0.013), the 2019 uACR (HR = 1.239, *p* = 0.034), and the 2020 uACR (HR = 1.444, *p* = 0.014) were independently associated with for MACE risk. In addition, after adjusting for potential confounders, the HRs for MACE risk were 1.145 (*p* = 0.132), 1.337 (*p* = 0.007), and 1.648 (*p* = 0.009) in the moderate-stable, high-stable, and elevated-increasing groups, respectively. The HRs for MACE risk were 1.203 (*p* = 0.028), 0.872 (*p* = 0.024), 1.562 (*p* = 0.033), and 2.218 (*p* = 0.002) in the moderate-stable, moderate-decreasing, high-stable, and elevated-increasing groups, respectively (**Table 3**).

**Table 3:** Multivariate Cox proportional hazards model.

Variable	*β*	SE	*χ* ^2^	HR	95% CI	*p*-value
Age (years)	0.435	0.186	2.347	1.545	1.073–2.386	0.019
DM course	0.363	0.124	2.928	1.436	1.127–2.385	0.003
HbA1c	0.439	0.167	2.631	1.548	1.119–2.385	0.008
HbA1c trajectory						
Low-stable	Reference					
Moderate-stable	0.186	0.083	2.276	1.203	1.026–1.411	0.028
Moderate-decreasing	0.137	0.061	2.252	0.872	0.774–0.983	0.024
High-stable	0.446	0.213	2.110	1.562	1.030–2.369	0.033
Elevated-increasing	0.801	0.255	3.126	2.218	1.346–3.659	0.002
NT-proBNP	0.568	0.041	2.755	1.764	1.178–2.386	0.006
LVMI	0.377	0.017	2.923	1.461	1.133–2.385	0.005
SGLT-2i	0.184	0.074	2.516	0.832	0.721–2.385	0.013
uACR (2019)	0.215	0.103	2.168	1.239	1.016–1.512	0.034
uACR (2020)	0.368	0.145	2.473	1.444	1.079–1.936	0.014
uACR trajectory						
Low-stable	Reference					
Moderate-stable	0.136	0.078	1.506	1.145	0.960–1.367	0.132
High-stable	0.291	0.108	2.704	1.337	1.083–1.652	0.007
Elevated-increasing	0.504	0.189	2.673	1.648	1.139–2.387	0.009

HR, hazard ratio; DM, diabetes mellitus; HbA1c, hemoglobin A1c; NT-proBNP, N-terminal pro-B-type natriuretic peptide; LVMI, left ventricular mass index; SGLT-2i, sodium–glucose co-transporter 2 inhibitor; uACR, urinary albumin/creatinine ratio.

### 3.5 Predictive value of the uACR and HbA1c trajectories for MACE

**Figure 2:**
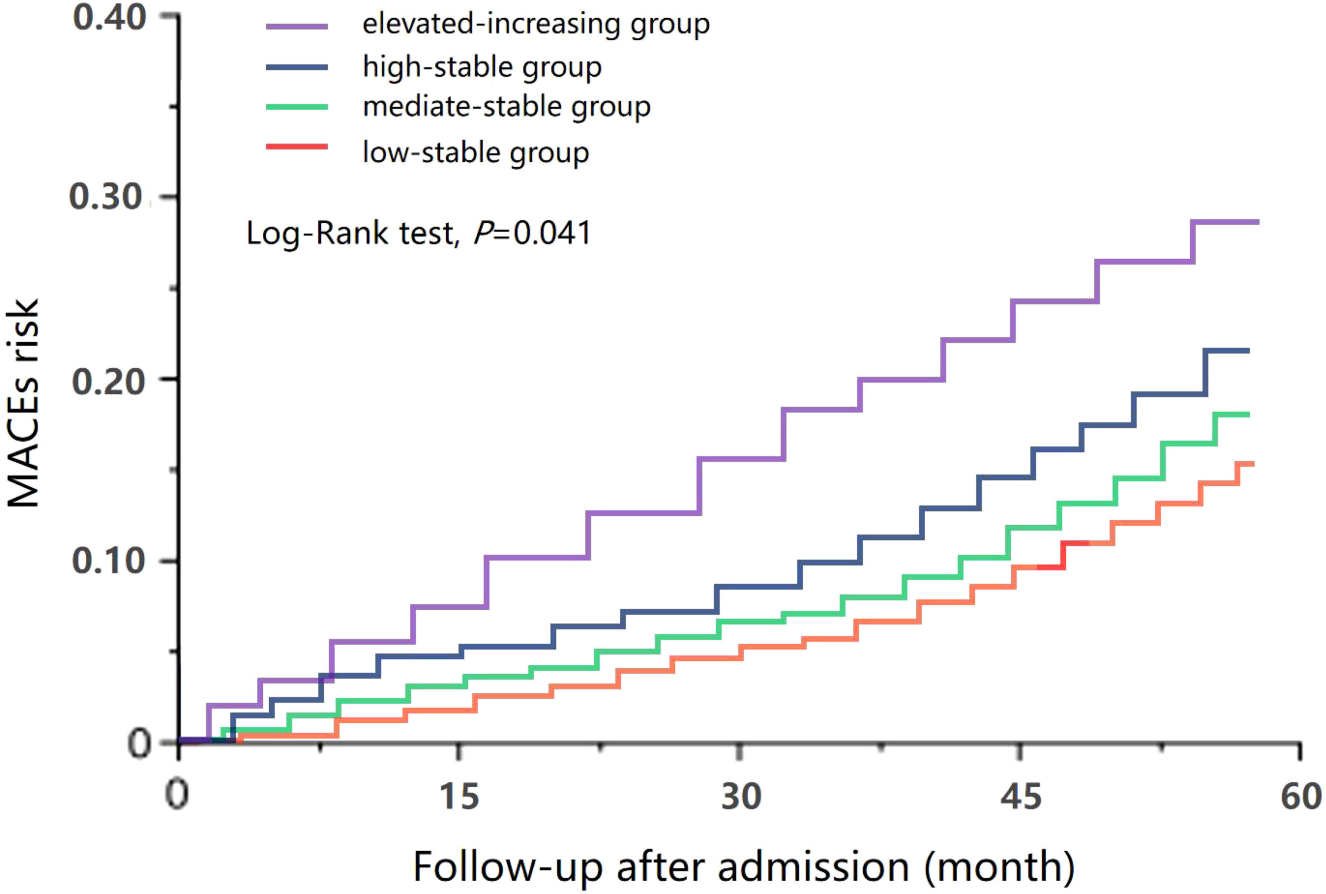
Receiver operating characteristic (ROC) curve of major adverse cardiac event (MACE) risk prediction using the model before and after adding urinary albumin/creatinine ratio (uACR), hemoglobin A1c (HbA1c), or both trajectories.

A predictive model for MACE was formulated based on the outcomes of the multivariate Cox regression analysis. ROC curve analysis demonstrated a notable enhancement in the accuracy of the model after the addition of uACR, HbA1c, or both trajectories. Specifically, before adding uACR or HbA1c, the AUC was 0.741; however, after adding uACR, HbA1c, or both, the AUCs were 0.773, 0.792, and 0.826, all of which signified statistically significant improvements (*p* = 0.021, 0.035, and 0.019, respectively) (**Figure 2**).

## 4 Discussion

After including 601 patients with T2DM admitted at our hospital, we found an important impact of the longitudinal patterns of uACR on the risk of MACE in diabetic patients. Compared with that in the low-stable group, the incidence of MACE in the high-stable group (HR = 1.337, 95% CI = 1.083–1.652, *p* = 0.007) and in the elevated-increasing group (HR = 1.648, 95% CI = 1.139–2.387, *p* = 0.009) significantly increased. Similar results were observed for HHF, non-fatal AMI, and acute renal injury/dialysis indications (*p* < 0.05). Therefore, our study demonstrated that a long-term elevated-increasing uACR is associated with a significantly increased risk of MACE in patients with diabetes, and regular monitoring of uACR could help identify diabetic patients with a higher risk of MACE.

There is a strong relationship between uACR and the prognosis of patients with T2DM. An increase in the uACR level is a strong predictor of cardiovascular events or death. Previous studies have shown that the uACR is an independent marker of systemic vascular endothelial dysfunction (14). In another retrospective study with a total of 66,311 patients with T2DM who had no prior history of cardiovascular disease, a linear positive correlation between uACR and the risk of cardiovascular disease or death was found during follow-up (*p* < 0.05). Furthermore, compared with the normal proteinuria group, the risks of cardiovascular disease (HR = 1.58) and death (HR = 2.08) in the microalbuminuria group were significantly increased (15). In addition, Wang et al. explored the relationship between uACR and acute ischemic stroke and found that uACR is an independent risk factor for ischemic stroke in participants without diabetes, hypertension, or cardiovascular disease (*p* < 0.05) (16). In this study, the 2019 uACR and the 2020 uACR were also shown to be important risk factors for MACE risk in patients with T2DM (*p* < 0.05).

However, only a few studies have evaluated the impact of the uACR trajectory on the prognosis of patients with diabetes. In patients with CKD, Cohen et al. found that the longitudinal trajectory of renal function was associated with cardiovascular events (17). The authors analyzed 2,438 patients with CKD in the CRIC study and found that, for every 8 mL min^−1^ 1.73 m^−2^ per year increase in the estimated glomerular filtration rate (eGFR), the risk of heart failure increased by 1.28 times and that of MACE increased by 1.11 times. For every 240 mg g^−1^ year^−1^ increase in uACR, the risk of heart failure increased by 1.20 times and that of MACE increased by 1.12 times (17). Another recent study explored the patterns of renal function changes in non-CKD populations and their association with cardiovascular outcomes (18), which included a total of 23,760 participants (average age, 58.63 years). During a follow-up period of 20.56 years, 8,328 patients (35.05%) had MACE. The researchers identified four eGFR trajectories and three patterns of CKD progression. Compared with the subjects in class I (high to mild eGFR decline group), those in class II (normal to mild eGFR decline group), class III (normal to moderate eGFR decline group), and class IV (mild to severe eGFR decline group) had adjusted odds ratios (ORs) for MACE risk of 1.11 (95% CI = 1.01–1.23), 1.27 (95% CI = 1.14–1.40), and 1.56 (95% CI = 1.38–1.77), respectively. Similarly, compared to those in the stable group, the HRs of the MACEs in the renal function slow progression group and the rapid progression group were significantly increased [1.75 (95% CI = 1.39–2.21) and 2.19 (95% CI = 1.68–2.86), respectively]. In the CARDIA study, which included 2,647 participants aged 18–30 years, the authors used latent class modeling to determine the trajectories of uACR from the year 10 examination to the year 30 examination. They identified five trajectory groups of uACR, namely, the low-stable group (64.9%), the moderate-stable group (25.8%), the high-stable group (4.4%), the moderate-increasing group (3.3%), and the high-increasing group (1.6%). They found that dynamic changes in the uACR were independently associated with adverse alterations in the cardiac structure and the LV systolic and diastolic functions (19). In another study that enrolled 329 patients with biopsy-proven diabetic kidney disease, the authors used joint latent class mixed models and identified three trajectory groups of uACR: the high-increasing group (77.2%), the high-decreasing group (7.3%), and the low-stable group (15.5%) (7). During the first 2 years of follow-up, it was confirmed that dynamic changes in the uACR were associated with subsequent end-stage kidney disease and all-cause mortality (*p* < 0.05). Comparable to previous results, our findings also confirmed that, compared with that in the low-stable group, the incidence of MACE in the high-stable group (HR = 1.337, 95% CI = 1.083–1.652, *p* = 0.007) and the elevated-increasing group (HR = 1.648, 95% CI = 1.139–2.387, *p* = 0.009) significantly increased. Therefore, the uACR trajectory is also an independent risk factor for MACE and long-term elevated-increasing uACR is associated with a significantly increased risk of MACE in the T2DM population.

Combining uACR with other markers could increase the accuracy of prognostic prediction. eGFR has been proven to be an important indicator of renal function grading; hence, the combination of uACR and eGFR can further increase the predictive accuracy for early renal function impairment. Fung et al. found that in male patients with T2DM, a concomitant uACR of 1–1.4 mg/mmol and an eGFR ≥90 mL min^−1^ 1.73 m^−2^ were associated with a significantly increased risk of MACE (HR = 1.25). In female patients, a concomitant uACR of 2.5–3.4 mg/mmol and an eGFR ≥90 mL min^−1^ 1.73 m^−2^ were associated with a markedly increased MACE risk (HR = 1.45) (15). Gerstein et al. reanalyzed the REWIND study that enrolled 9,901 patients with T2DM. During a median follow-up of 5.4 years, renal outcomes developed in 848 (17.1%) patients. The authors developed a novel prognostic indicator that combined uACR and eGFR, called the kidney disease index (KDI). The primary outcome was to evaluate the baseline levels of 1/eGFR and natural log-transformed uACR (calculated as ln[uACR × 100]) and their interactions for MACE, kidney outcomes, and death (20). The study found a nonlinear association between 1/eGFR and all three prognoses and between ln[uACR × 100] and kidney outcomes, but a negative relationship between 1/eGFR and uACR and MACE. Furthermore, there was a linear relationship between the KDI and all three outcomes. The *C* statistics for the KDI were comparable to those for uACR and eGFR (20).

In addition, adding uACR to the risk model can increase the predictive accuracy of the prognosis. In a recently published retrospective cohort study that enrolled 632 aged diabetic patients, Liu et al. found that, as uACR increased, the risk of new-onset heart failure (NOHF) gradually increased, as shown in the restricted cubic spline curve (*p*_trend_ < 0.05). After adding uACR, the ROC curve showed significant improvement in the accuracy of predicting NOHF (AUC = 0.692 and 0.785, respectively, *p* < 0.001). Furthermore, the addition of uACR was also associated with a significant improvement in the classification of NOHF, with a net weight classification improvement (net reclassification improvement, NRI) of 0.343 (*p* = 0.006) and an integrated discrimination improvement (IDI) of 0.032 (*p* = 0.001) (21). Tao et al. added uACR to a heart failure risk model (WATCH-DM) and found that the new model (WATCH-DM + uACR) was associated with a significantly increased predictive ability for MACE risk (AUC = 0.744 *vs.* 0.802), as well as the NRI (*p* < 0.05) and IDI (*p* < 0.05) (22). Therefore, the addition of uACR can improve MACE risk prediction in patients with T2DM and can significantly improve a patient’s prognosis classification.

## 5 Limitations

The present study has several limitations.

1) This is a single-center retrospective study with a small sample size. The research results may differ from those of other research centers.
2) Only patients with uACR ≤ 300 mg/g were included in this study. Those with a higher uACR (>300 mg/g) have been proven to be associated with a significantly increased risk of MACE; therefore, these participants were excluded.
3) We used latent mixed modeling to identify four distinct trajectories of uACR: the low-stable group, moderate-stable group, high-stable group, and elevated-increasing group. However, in the clinic, some patients (<10%) have shown a reduction in uACR and a decreased risk of MACE. In this study, we did not find this subgroup owing to the small volume (23).
4) The limited number of uACRs monitored during the exposure period in this study could have led to misjudging the change in trajectories and could have affected the final results. A high-quality prospective study with a large sample size is needed to confirm the findings of this study.
5) The baseline data for the uACR trajectories were not comparable. In particular, several important variables that have a significant impact on the occurrence of MACE, such as age, DM course, the levels of HbA1c, NT-proBNP, and LVMI, and the patients treated with proportion of β-receptor blockers, did not match among the four groups. We are currently continuing this study, and, as more patients are included, we will use propensity score matching methods to match the baseline data between groups and minimize the impact of baseline data on prognosis.
6) The median follow-up was only 45 months. A longer follow-up is needed as the occurrence of MACE is expected to accrue over time.

## 6 Conclusions

A long-term elevated-increasing uACR is associated with a significantly increased risk of MACE in patients with diabetes. The results of this study imply that regular monitoring of uACR could be helpful in identifying diabetic patients with a higher risk of MACE. However, this finding must be interpreted with caution as the evidence supporting the use of a longitudinal uACR trajectory to predict the risk of MACE is limited by the small sample size and the short follow-up course of previous studies. Large, high-quality studies are warranted to confirm the predictive value of the longitudinal uACR trajectories for MACE risk in patients with T2DM, especially in those at high risk for cardiovascular diseases.

## Data availability statement

The datasets used and/or analyzed during the current study are available from the corresponding author on reasonable request.

## Ethics statement

The studies involving humans were approved by the Ethics Committee of First Hospital of Shanxi Medical University and Second Hospital of Shanxi Medical University. The studies were conducted in accordance with local legislation and institutional requirements. The ethics committee/institutional review board waived the requirement of written informed consent for participation from the participants or the participants’ legal guardians/next of kin because this was a retrospective study.

## Author contributions

HL: Writing – original draft, Data curation. YR: Investigation, Data curation, Writing – original draft, review & editing. YD: Data curation, Writing – review & editing. PL: Data curation, Writing – original draft. YB: Conceptualization, Writing – review & editing.
